# Validating lane drifts as a predictive measure of drug or sleepiness induced driving impairment

**DOI:** 10.1007/s00213-019-05424-8

**Published:** 2020-01-03

**Authors:** F. R. J. Vinckenbosch, A. Vermeeren, J. C. Verster, J. G. Ramaekers, E. F. Vuurman

**Affiliations:** 1 Department of neuropsychology and psychopharmacology, Maastricht University, Maastricht, The Netherlands; 2 Institute for Risk Assessment Sciences, Utrecht University, Utrecht, The Netherlands; 3 Division of Pharmacology, Utrecht University, 3584CG, Utrecht, The Netherlands; 4 Centre for Human Psychopharmacology, Swinburne University, Melbourne, Australia

**Keywords:** Drugs, On-the-road driving, SDLP, Lane drifts, Lapses of attention

## Abstract

**Background:**

Standard deviation of lateral position (SDLP) has been accepted as a reliable parameter for measuring driving impairment due to lowered vigilance caused by sleepiness or the use of sedating drugs. Recently, lane drifts were proposed as an additional outcome measure quantifying momentary lapses of attention. The purpose of this study was to validate lane drifts as outcome measure of driver impairment in a large data pool from two independent research centers.

**Methods:**

Data from 11 placebo-controlled studies that assessed the impact of alcohol, hypnotics, and sleep deprivation on actual driving performance were pooled. In total, 717 on-the-road tests performed by 315 drivers were subjected to an automated algorithm to detect occurrences of lane drifts. Lane drifts were defined as deviations > 100 cm from the mean (LD_mlp_) and from the absolute lateral position (LD_alp_) for 8 s.

**Results:**

The number of LD_mlp_ was low and did not differ between treatments and baseline, i.e., 14 vs. 3 events, respectively. LD_alp_ were frequent and significantly higher during treatment relative to baseline, i.e., 1646 vs. 470 events. The correlation between LD_alp_ and SDLP in the treatment conditions was very high (r_s_ = 0.77). The frequency of the occurrence of treatment-induced lane drifts however depended on baseline SDLP of drivers, whereas treatment-induced changes in SDLP occurred independent of baseline SDLP.

**Conclusion:**

LD_mlp_ is not useful as an outcome measure of driver impairment due to its rare occurrence, even when treatment-induced increments in SDLP are evident. Treatment effects on LD_alp_ and SDLP are closely related.

## Introduction

Sustained attention is a necessary requirement for the safe operation of a motor vehicle in traffic. It has been estimated that up to 15% of the traffic accidents on motor ways are associated with sleepiness (Maycock, 1996). CNS drugs such as hypnotics are known to produce sleepiness and may affect psychomotor and executive function (Jongen et al., 2018). Consequently, epidemiological studies have repeatedly demonstrated a positive association between the use of sedative medications and accident risk (Barbone et al., 1998; Movig et al., 2004; Gustavsen et al., 2008; Orriols et al., 2011).

For over 30 years, the “gold standard” for the assessment of the effects of drugs and sleepiness on driver vigilance in experimental, placebo-controlled studies has been a standardized on-the-road driving test (Ramaekers, 2017) that was developed in the Netherlands. This naturalistic test requires participants to complete a 100-km test drive on a primary highway while accompanied by a licensed driving instructor who has access to dual controls. The participants are instructed to maintain a steady lateral position in the middle of the right traffic lane at a velocity of 95 km/h. During the drive, a camera mounted on top of the vehicle continuously monitors the vehicle’s lateral position relative to the traffic lane demarcation to the left of it.

The standard deviation of lateral position (SDLP), i.e., the weaving of the car, is the main outcome measure of the standardized driving test. It is considered to be a quantification of lane weaving, a measure of vehicle control. It has been repeatedly demonstrated that mean SDLP increases with approximately 2.5 cm when driving under a blood alcohol concentration (BAC) of 0.5 g/L, relative to driving under placebo. A BAC of 0.5 g/L is the legal limit for driving under the influence of alcohol in most European countries, because epidemiological studies have demonstrated that crash risk increases at concentrations exceeding this threshold (Borkenstein et al., 1974). Consequently, an increase in SDLP of 2.5 cm or higher represents a clinically relevant change in the on-the-road driving test when quantifying medicinal drug effects on SDLP. On-the-road driving studies have demonstrated that the use of sedative medications, such as benzodiazepines and antidepressants, and sleep deprivation can produce increments in SDLP that are equal to or greater than this clinical threshold (Jongen et al., 2018; Veldhuijzen et al., 2006; Ramaekers et al., 1998; Jongen et al., 2015). The implication is that these challenges can increase crash risk, similar to alcohol. Drug- and alcohol-induced changes in SDLP observed in the on-the-road driving tests are strongly correlated to drug- and alcohol-induced crash risk as assessed in epidemiological studies (Ramaekers, 2017). The implication is that SDLP as measured in the on-the-road driving test is not merely a measure of driver impairment but also predicts crash risk.

However, despite the agreement that the SDLP is sensitive measure for driver vigilance, it has been argued that the association between drug- and sleepiness-induced increments in SDLP and crash risk is indirect (Lococo & Staplin, 2006; Hartman et al., 2015; Verster et al., 2014) and that other factors may play a more important role in the occurrence of an accident such as brief moments of inattention, micro-sleeps, and distraction (Verster et al., 2014; Verster et al., 2018; Verster & Roth, 2014a). Two additional, potentially relevant, measures for predicting crash risk have been proposed, i.e., lane excursions and lane drifts, which are both derived from the same source parameter as SDLP, i.e., lateral position. Lane excursions were quickly discarded as a sensitive measure of crash because they occur infrequently and are much less sensitive than SDLP for demonstrating driver impairment (Verster & Roth, 2014a). Lane drifts however have been proposed as a valuable measure of lapses of attention that may occur during prolonged driving (Verster et al., 2014). The authors who proposed this measure reanalyzed driving data from two double-blind, placebo-controlled on-the-road driving studies that examined the residual effects of hypnotic drugs on lateral position. The reanalysis showed that both SDLP and lapses of attention were significantly higher following administration of hypnotics as compared to placebo. A lapse of attention in this context was conceptualized by the authors as “a short period of inattention during which the driver experiences reduced alertness and does not focus on the task, or actually stops performing the task, resulting in driving impairment”. It was argued that a momentary reduction or loss of attention would lead to a relatively large deviation in lateral position. Hence, a lapse of attention during the on-the-road driving test is operationalized as “a deviation from mean lateral position of more than 100cm for four or more seconds” (Verster et al., 2014). Post hoc, the lapse duration criterion was increased to 8 s since it was found that only 4.6% of the observed deviations of > 100 cm had a duration of 4 to 8 s (Verster et al., 2014). In a later publication, an alternative operationalization was applied on the same dataset that defined a lapse “as a continuous change in lateral position of greater than 100 cm, lasting for at least 8 seconds” (Verster et al., 2018). Both operationalizations were reported to be able to detect a significant increase in the number of lane drifts during on-the-road driving in the morning after nighttime administration of hypnotics such as zolpidem, zopiclone, and ramelteon (Verster et al., 2014; Verster et al., 2018). It was therefore concluded that lane drifts are a useful outcome measure of driving impairment during on-the-road driving that is conceptually distinct from driver impairments assessed with SDLP. So far, lane drifts as a measure of driving impairment have been piloted in two datasets from on-the-road driving studies (Verster et al., 2014; Verster et al., 2018; Mets et al., 2011) as described above. In addition, the outcome has been applied in a study on the effects of methylphenidate on the driving performance of ADHD patients where it was found that treatment significantly reduces the number of lane drifts (Verster & Roth, 2014b). Another study into the effects of the orexin antagonist Lemborexant in healthy volunteers did not find any lane drifts at all, which is in contrast to the aforementioned findings which state that lane drifts were also apparent after placebo treatment in healthy volunteers (Verster et al., 2014). A large-scale validation of the measure’s sensitivity to drug-induced impairment and how it discriminates from SDLP is still lacking. The purpose of this study was to further investigate and validate lane drifts as outcome measure of drug- and sleepiness-induced driver impairment in a large data pool from two independent research centers that included on-the-road driving data from 11 placebo-controlled studies that assessed the impact of alcohol, hypnotics, and sleep deprivation on actual driving performance.

## Methods

In order to validate proposed operationalizations of lane drifts (Verster et al., 2014; Verster et al., 2018), data was pooled from 11 randomized, placebo-controlled, cross-over studies that employed the on-the-road driving test and were conducted at two independent research centers. These studies were originally designed to assess the effects of alcohol (Kuypers et al., 2006; van der Sluiszen et al., 2016), hypnotics (Jongen et al., 2018; Mets et al., 2011; Leufkens et al., 2009; Vermeeren et al., 2018; Vermeeren et al., 2016; Vermeeren et al., 2015; Vermeeren et al., 2014; Verster et al., 2002), and sleep deprivation (Jongen et al., 2015) and were selected for the current investigation because the administered treatments were found to significantly affect the SDLP. The pooled dataset included 315 healthy volunteers of both sexes who drove after alcohol (*N* = 49), zopiclone (*N* = 194), zolpidem (*N* = 72), oxazepam (*N* = 43), diazepam (*N* = 21) and sleep deprivation (*N* = 23) and after placebo (*N* = 315). For an overview of the studies included samples sizes per study, references, and research center, see Table 1. For studies where the effect of a substance was assessed at two successive time points, only the first measurement was included. It is noted that for two studies (Mets et al., 2011; Verster et al., 2002), the number of participants differs from the original publications because the data of 11 driving tests could not be recovered.

**Table 1:** Summary of included data. The time of the start of the driving test (tstart driving test) is relative to the time of drug administration

Study	Treatment	tstart driving test	Research center
Kuypers et al. (2008)	Alcohol 0.5 g/L (*N* = 18)	+ 2 h	Maastricht University
	Placebo (*N* = 18)	+ 2 h	
van der Sluiszen et al. (2016)	Alcohol 0.5 g/L (*N* = 31)	+ 1.5 h	Maastricht University
	Placebo (*N* = 31)	+ 1.5 h	
Vermeeren et al. (2014)	Zopiclone 7.5 mg (*N* = 40)	+ 9 h	Maastricht University
	Placebo (*N* = 40)	+ 9 h	
Vermeeren et al. (2015)	Zopiclone 7,5 mg (*N* = 28)	+ 9 h	Maastricht University
	Placebo (*N* = 28)	+ 9 h	
Vermeeren et al. (2016)	Zopiclone 7.5 mg (*N* = 24)	+ 9 h	Maastricht University
	Placebo (*N* = 24)	+ 9 h	
Vermeeren et al. (2018)	Zopiclone 7.5 mg (*N* = 48)	+ 9 h	Maastricht University
	Placebo (*N* = 48)	+ 9 h	
Mets et al. (2011)	Zopiclone 7.5 mg (*N* = 29)	+ 8.5 h/10 h	Utrecht University
	Placebo (*N* = 29)	+ 8.5 h/10 h	
Leufkens et al. (2009)	Zopiclone 7.5 mg (*N* = 25)	+ 10 h	Maastricht University
	Zolpidem 10 mg (*N* = 24)	+ 5 h	
	Placebo (*N* = 25)	+ 5 h/+ 10 h	
Verster et al. (2002)	Zolpidem 10 mg (*N* = 23)	+ 4 h	Utrecht University
	Zolpidem 20 mg (*N* = 25)	+ 4 h	
	Placebo (*N* = 27)	+ 4 h	
Jongen et al. (2018)	Diazepam 10 mg (*N* = 21)	+ 4 h	Maastricht University
	Oxazepam 30 mg (*N* = 22)	+ 4 h	
	Oxazepam 10 mg (*N* = 21)	+ 4 h	
	Placebo (*N* = 22)	+ 4 h	
Jongen et al. (2015)	Sleep deprivation (*N* = 23)	–	Maastricht University
	Normal sleep (*N* = 23)		

For every test drive, SDLP was calculated as the standard deviation relative to the mean lateral position over the entire 100-km drive, as opposed to the average of the SDLP across 20 successive segments of 5 km, which is traditionally employed (O'Hanlon et al., 1982; Ramaekers et al., 1992; O'Hanlon, 1984). The segmented approach corrects for drifts in the mean lane position over time that frequently occur and may lead to inflated SDLP values. Still, it was opted to calculate SDLP from the mean lateral position over the entire test drive to eliminate differences in the calculation of SDLP between the two centers. The Maastricht center traditionally uses the segmented approach for calculating SDLP, whereas the Utrecht center treats driving data over 100 km as a single segment (Verster & Roth, 2011). Consequently, mean SDLP values from studies conducted in the Maastricht research center will be higher as those reported in their original publications.

**Fig. 1:**
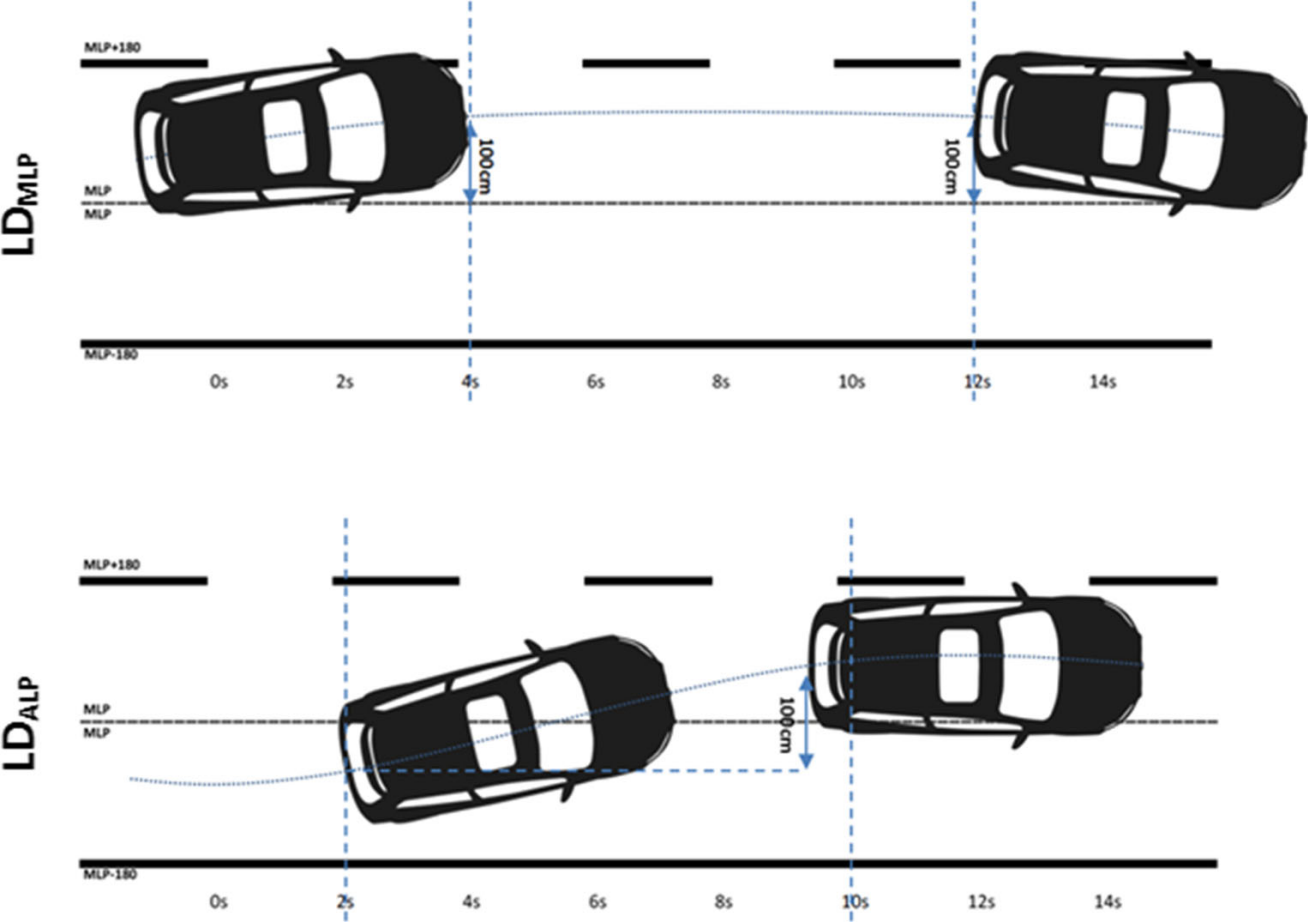
Illustrations depicting a lane drift relative to the mean lateral position (LDmlp) and lane drifts relative to the absolute lateral position (LDalp)

In previous publications (Verster et al., 2014; Verster et al., 2018), lane drifts were evaluated and classified by visual inspection of the individual driving data. In order to control for interrater differences and to objectify the scoring procedure, an automated algorithm was created to detect occurrences of lane drifts in all individual drives. Lane drifts were defined as deviations > 100 cm from the mean (LD_mlp_) and from the absolute lateral position (LD_alp_) for 8 s as previously suggested (Verster et al., 2014; Verster et al., 2018) (Fig. 1). The former essentially defines a lane drift as the event where a driver crosses the lane border for more than 8 s, whereas the latter qualifies a lane drift as any lateral displacement > 100 cm that occurs within a time window of 8 s. The automated algorithm was programmed to calculate mean SDLP and to count the number of lane drift according to the two definitions given above.

### Statistical analysis

For the statistical analysis, participant data was grouped per treatment. First, in order to assure that the method of calculating the SDLP, i.e., as the standard deviation of the entire test as opposed to the average of the standard deviations per 5-km segment, did not affect the relative drug effects reported in the original investigations, the Pearson correlation was determined between the two calculation methods for the largest group in the sample, i.e., participants treated with zopiclone 7.5 mg and placebo (*N* = 194), but not for all available test drives since the aggregation and parallel analysis of all datasets is time costly and was not considered necessary to make this minor point. Next, in order to investigate whether lane drifts are indeed sensitive measures for observing driving impairment, the statistical significance of the difference in SDLP values between each treatment and the respective baseline condition was tested with a paired samples t-test. For the statistical testing of the difference in number of LD_mlp_ and LD_alp_ between the treatment and baseline conditions, the non-parametric Wilcoxon signed rank test was selected because of the severe skewedness of the data which could not be resolved by logarithmic transformations. In addition, the correlation between the SDLP and number of LD_alp_ in the treatment conditions, and the correlation between the increase in SDLP (ΔSDLP) and the number of lane drifts was determined. It was hypothesized that if lane drifts are a true measure of driving impairment rather than a mere transformation of the SDLP, the relationship between the number of LD and the ΔSDLP should be stronger and more consistent than the relationship between the LD and the absolute SDLP values during treatment, considering that it is the increase in SDLP which conveys information about driving impairment rather than the absolute SDLP value. If the correlation between the lane drifts and absolute SDLP values are stronger, this suggests that lane drifts are a mere transformation of SDLP rather than a measure of driving impairment. Because the scatterplots suggested a quadratic pattern for both relationships, Spearman’s *ρ*(r_s_) was chosen over Pearson’s *r*. Lastly, in order to further investigate the independence of lane drifts from the SDLP, the Spearman correlation was calculated between the baseline values of the SDLP of individuals and their ΔSDLP during treatments, as well as the correlation between the baseline SDLP values of individuals and their ΔLD_alp_ during treatment. It is to be expected that neither the ΔSDLP nor the ΔLD_alp_ correlates significantly with the baseline SDLP since the variation in the baseline SDLP is not assumed to reflect driving performance or driver fitness. For all statistical tests, the significance level was set at α = 0.05. All statistical analyses were conducted using the statistical package for the social sciences (*SPSS) edition 24* offered by IBM.

## Results

**Fig. 2:**
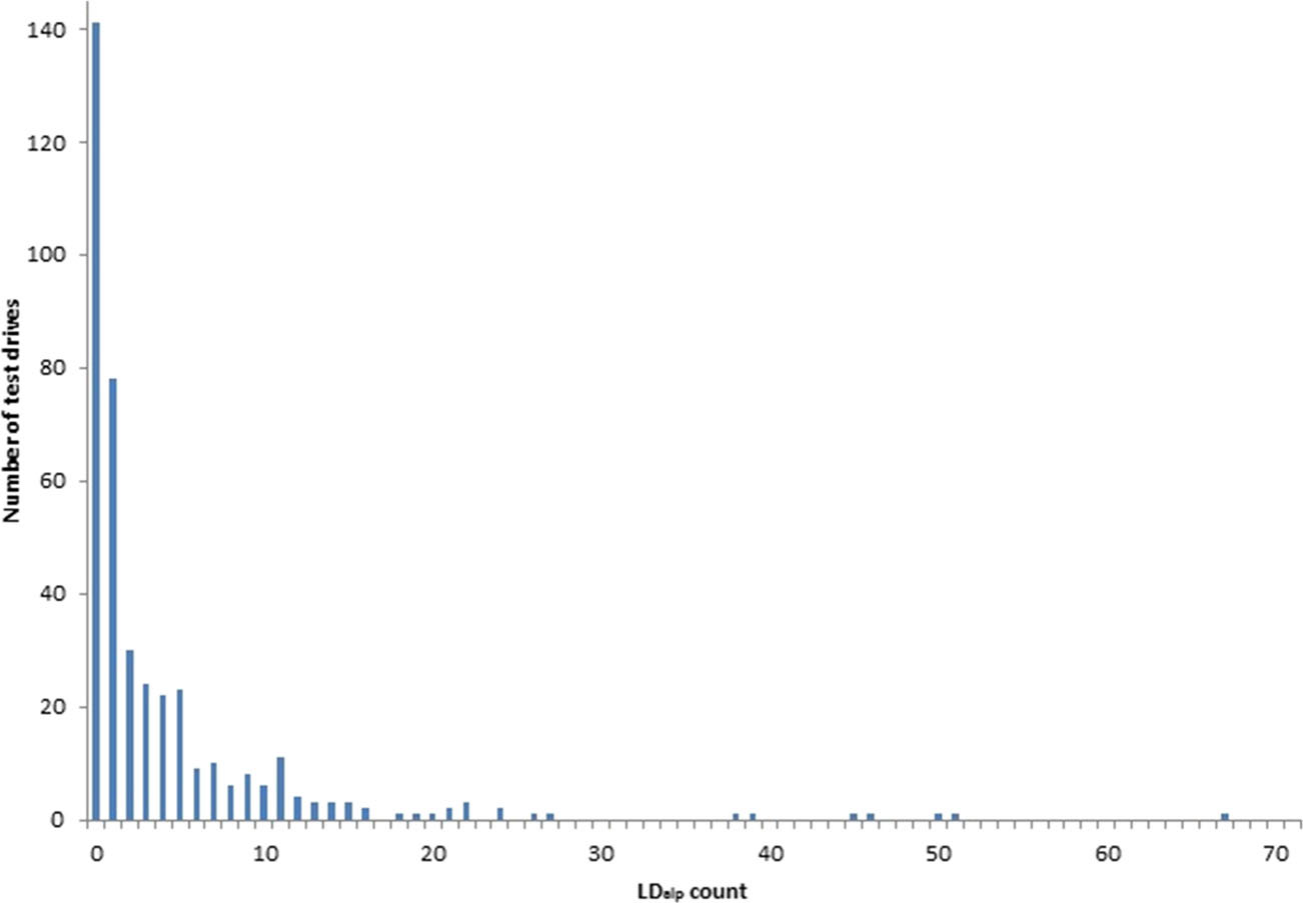
Histogram of the number of lane drifts relative to the absolute lateral position (LDalp) during the treatment conditions (*N* = 402)

The Pearson correlation between SDLP values calculated from the mean lateral position over the entire ride and across segments was highly significant for the 194 zopiclone 7.5 mg drives (*r*[163] = 0.84, *p* < 0.001), as well as for the 194 corresponding baseline drives (*r*[163] = 0.89, *p* < 0.001). For all treatment conditions, a significant increase in SDLP was observed compared to baseline. Overall, only 14 LD_mlp_ events were detected during treatment conditions, and only 3 LD_mlp_ events occurred during baseline. LD_mlp_ did not significantly differ between treatments and baseline. LD_alp_ events however occurred very frequently. A total of 1646 LD_alp_ events were established in the treatment conditions vs. 470 events during the baseline conditions. As can be seen in Fig. 2, the distribution was positively skewed, with the majority of test drives in the treatment conditions (54.5%) including no or a single LD_alp_ event. About 7.5% of the sample accounted for approximately 50% of all detected LD_alp_. Overall, the number of LD_alp_ was significantly higher during most treatments as compared to baseline, with the exception of the oxazepam 10 mg and diazepam 10 mg conditions.

**Fig. 3:**
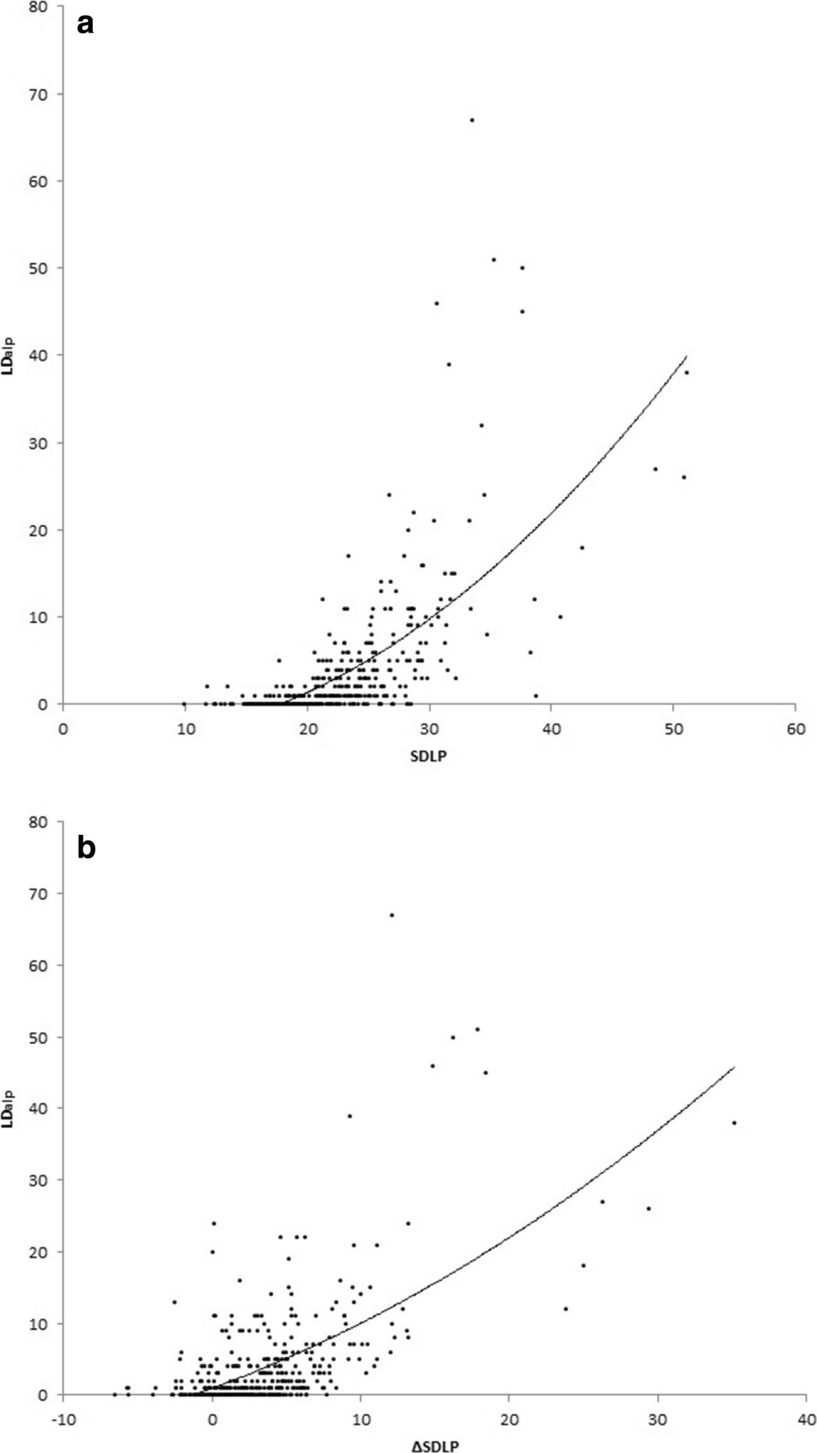
Scatterplots depicting the relationships between **[A]** the SDLP values and the number of lane drifts relative to the absolute lateral position(LDalp), and **[B]** the change in SDLP between placebo and treatment and the number of LDalp

The correlation analyses demonstrated a consistent and highly significant correlation between SDLP and the number of LD_alp_ after treatment (*rs*(400) = 0.77, *p* < 0.001). Also, the correlation between the ΔSDLP and the number of LD_alp_ during the treatment conditions was significant (*rs*(400) = 0.50, *p* < 0.001). Significant correlations between SDLP and number of LDalp and ΔSDLP and LDalp across treatment conditions are shown in Fig. 3.

For each treatment separately, correlations between SDLP and number of LD_alp_ in the treatment conditions were found to be significant as well as the correlation between the ΔSDLP and LD_alp_ with the exception of the alcohol condition. The correlation analysis of the relationship between the baseline SDLP values on the one hand and ΔSDLP and ΔLD_alp_ on the other only yielded a significant Spearman correlation between the baseline SDLP and the ΔLD_alp_ in the zopiclone 7.5 mg group (*rs*(192) = 0.20, *p* = 0.004), but no significant correlation between the baseline SDLP values and the ΔSDLP in this same treatment group (*rs*(192) = −0.11, *p* = 0.14). All other correlations were found to be insignificant. Table 2 provides a summary of the results.

**Table 2:** Summary of the results

Treatment	N	Mean SDLP (SD)	Mean ΔSDLP (SE) [a]	Lane drifts relative to MLP	Paired difference: +: − [_1_]	W+: W− [_2_] [b]	Lane drifts relative to ALP	Paired difference: +: − [_1_]	W+: W− [_2_][b]	rs(SDLP- LDALP) [c]	rs(ΔSDLP-LDALP) [c]	rs(SDLPpla- ΔSDLP) [c]	rs(SDLPpla- ΔLDALP) [c]
Alcohol	49	22.97 (5.19)	+ 2.65 (0.42)***	5	1: 0	5: 0	197	25: 10	455: 175*	0.826***	0.271	−0.019	−0.009
Alcohol placebo		20.32 (4.33)		0			137						
Zopiclone	194	21.92 (3.82)	+ 2.60 (0.19)***	1	1: 0	1: 0	413	94: 20	5424.5: 1130.5***	0.707***	0.422***	−0.106	0.204**
Zopiclone placebo		19.32 (3.82)		0			178						
Zolpidem 10 mg	47	23.10 (5.52)	+ 3.78 (0.68)***	5	3: 2	10: 5	305	25: 9	504.4: 90.5***	0.835***	0.585***	−0.120	0.145
Zolpidem placebo		19.37 (3.59)		3			85						
Zolpidem 20 mg	25	27.98 (11.23)	+ 9.86 (2.12)***	3	2: 1	3.5: 2.5	380	17: 5	227.5: 25.5**	0.849***	0.788***	0.157	0.089
Zolpidem placebo		18.12 (3.99)		2			47						
Oxazepam 10 mg	21	21.02 (4.57)	+ 1.62 (0.71)*	0	–	–	47	10: 5	82.5: 37.5	0.769***	0.488*	−0.238	−0.084
Oxazepam placebo		19.4 (4.17)		0			30						
Oxazepam 30 mg	22	27.01 (5.94)	+ 7.74 (1.17)***	0	–	–	138	20: 2	222.5: 30.5**	0.682***	0.611**	−0.281	−0.137
Oxazepam placebo		19.27 (4.11)		0			30						
Diazepam 10 mg	21	22.4 (1.01)	+ 3.28 (0.56)***	0	–	–	75	11: 3	82.5: 22.5	0.729***	0.512*	−0.117	0.213
Diazepam placebo		19.12 (0.91)		0			30						
Sleep deprivation	23	20.62 (3.87)	+ 3.36 (0.63)***	0	–	–	91	12: 3	114: 6**	0.863***	0.543**	−0.154	0.303

**[**_**1**_**]** Ratio of the number of positive and negative paired differences (treatment – placebo); **[**_**2**_**]** ratio of sum of positive (W+) and sum of negative (W−) differences;**[a]** paired samples t-test; **[b]** Wilcoxon signed rank test; **[c]** Spearman correlation; *p < 0.05; **p < 0.01; ***p < 0.001

## Discussion

The primary goal of the current investigation was to validate lane drifts as an outcome measure of drug- and sleepiness-induced driver impairment as proposed by Verster et al. (Verster et al., 2014; Verster et al., 2018). An automated algorithm determined the number of lane drifts relative to the mean lateral position with a duration of at least 8 s (LD_mlp_) and the number of lane drifts relative to the absolute lateral position within a time window of 8 s (LD_alp_) in a large data pool. This data pool contained data of 315 test drives after placebo administration or no treatment and 402 test drives after administration of a sedative substance or sleep deprivation. Over all 717 test drives, only 19 LD_mlp_ were detected. The number of LD_mlp_ was not found to be significantly more prevalent in the treatment conditions. A total of 2116 LD_alp_ were identified in all test drives. It was also found that the number of LD_alp_ was significantly higher in the treatment conditions with the exception of the oxazepam 10 mg and diazepam 10 mg conditions.

It appears that LD_mlp_ are rare events. As a result, the outcome measure is unable to significantly discriminate between any treatment and respective baseline condition, despite the evident increase of the SDLP after all treatments. It is therefore clear that ΔLD_mlp_ as an outcome measure has an inferior sensitivity, if any, to driving impairment compared to the ΔSDLP. In contrast to LD_mlp_, multiple LD_alp_ were detected in every condition. The events appeared relatively frequently. However, the majority of drivers exhibited no or only one LD_alp_ during treatment. The distribution of LD_alp_ was highly skewed with less than 10 % of the participants accounting for approximately half of all detected events. The number of LD_alp_ was nearly always significantly higher in the treatment than in the baseline conditions, with the exception of the oxazepam 10 mg and diazepam 10 mg conditions. The absence of an effect could be attributed to the fact that in these conditions two drives were prematurely terminated, resulting in less opportunity for lane drifts to occur. In contrast, the increase in SDLP was found to be significant in all treatment conditions which suggest that the ΔSDLP is more sensitive for the detection of driving impairment, even if the duration of the test is shortened. An inspection of the correlation coefficients in Table 2 demonstrates that there is a close positive relationship between the number of SDLP and the LD_alp_ in the treatment conditions, as well as between the ΔSDLP and the LD_alp_. The former relationship was overall found to be stronger and more consistent across different treatments. The close relationship between the outcome measures is not surprising given that they are both derived from the lateral position of the vehicle.

As mentioned, the relationship between the absolute SDLP and LD_alp_ was overall stronger than the relationship between the ΔSDLP and LD_alp_. However, if LD_alp_ is a true measure of driving impairment, it would be expected that a closer relationship should exist between the ΔSDLP and LD_alp_. The absolute SDLP value is known to differ considerably between healthy individuals treated with placebo, while the test-retest reliability of the SDLP has been found to be high (Verster & Roth, 2011). Hence, the SDLP can be considered as a driving characteristic that varies considerably between healthy individuals, but not within, and therefore conveys little information about driving impairment per se. For this reason, it is the increase in SDLP (ΔSDLP) relative to placebo or baseline performance that is used in on-the-road driving studies to assess driving impairment. The close relationship between the absolute SDLP and LD_alp,_ while the relationship between the ΔSDLP and the LD_alp_ was found to be less strong and less consistent, suggests that LD_alp_ is simply a transformation of the SDLP rather than an independent measure of driving impairment. The absence of a correlation between baseline SDLP and treatment-induced ΔSDLP and the presence of a significant correlation between baseline SDLP and a treatment (zopiclone)-induced ΔLD_alp_ support this notion. It demonstrates that the ΔSDLP is independent from the baseline SDLP, while detection of LD_alp_ is biased toward participants with higher baseline SDLP values. However, for LD_alp_ to be a true measure of driving impairment, the increase in LD_alp_ should occur independent from the baseline SDLP value. The observation that this is not the case is problematic.

Consideration of the statistical nature of the SDLP can explain why the relationship between the absolute SDLP and the LD_alp_ count is stronger than the relation between the ΔSDLP and ΔLD_alp_, as well as provide an explanation for the modest correlation between the baseline SDLP and the increase in LD_alp_. The standard deviation of the lateral position is a parameter of the width of the confidence interval of the lateral position. It can be calculated that the 99% confidence interval of the lateral position is about 100-cm wide when a participant drives with a mean SDLP of 19.45 cm. As a result, participants with a SDLP under 19.45 cm would likely exhibit no LD_alp_, i.e., never produce a lane drift that spans more than 100 cm, despite the possibility of experiencing significant road tracking impairment as indicated by the SDLP. Therefore, the relationship between the absolute SDLP and LD_alp_ count is stronger than the relation between ΔSDLP and LD_alp_. This also means that participants with a low baseline SDLP of, e.g., 16 cm, should show greater impairment in road tracking ability as quantified by the ΔSDLP during the treatment condition than a participant with a high baseline SDLP of, e.g., 19 cm, in order to exceed the threshold of 19.45 cm. Taken together, the high correlation between the absolute SDLP and absolute LD_alp_ count and the statistical bias toward participants with higher inherent SDLP values suggest that LD_alp_ is a linear transformation of the SDLP that conceivably adds no new information and is less sensitive to impairment than the ΔSDLP.

Besides the abovementioned problems with the LD_mlp_ and LD_alp_ as parameters of driving impairment, a more fundamental issue remains. Verster et al. (Verster et al., 2014; Verster et al., 2018; Verster & Roth, 2014a) proposed lane drifts as a measure of momentary lapses of attention which were defined as “short periods of inattention during which the driver experiences reduced alertness and does not focus on the task, or actually stops performing the task, resulting in driving impairment”. However, it is uncertain whether a momentary lapse of attention leads to a significant change in lateral position. Of course, it appears likely that a significant lateral displacement would occur if the participant stops performing the task altogether. However, an event like this can arguably not be conceived as a lapse of attention, rather than falling asleep or losing consciousness. A lapse of attention is usually conceptualized as moment during which internal task irrelevant information is being processed at the cost of the processing of incoming external information and is often referred to as task unrelated thought (TUT) or mind wandering (Smallwood et al., 2004). A frequently employed lab task for the assessment of attentional lapses in this sense is the sustained attention to response task (SART). During this task, participants are instructed to respond to stimuli which are presented at a rapid pace and to withhold their response whenever a relatively infrequently presented target stimulus appears. A lapse is defined as a commission error, i.e., responding to the target stimulus which requires inhibition of a response. It has been argued that commission errors during the SART are the result of poor top-down motor control (motor decoupling), rather than inattention to external stimuli (perceptual decoupling) (Head & Helton, 2013), or both (Seli, 2016). Whatever the case, it is clear that higher level functioning can be impaired while lower level functioning, i.e., indiscriminant and automated responding to any stimulus, remains intact. Arguably, for experienced drivers, road tracking is also a highly automated skill which requires little focused attention. It is therefore reasonable to assume that a short period of inattention does not necessarily result in a measurable change in lateral position. Future research should adopt established physiological and behavioral measures of attention in order to assess if and when drivers experience attentional lapses and whether this is reflected in the lateral position of the vehicle.

## Conclusion

Lane drifts relative to the mean lateral position (≥ 100 cm) which last for at least 8 s are not a useful outcome measure of drug- and sleepiness-induced driving impairment. Due to the rare occurrence, it is unable to demonstrate driving impairment in various treatment conditions despite SDLP showing significant treatment-induced increases. Lane drifts relative to the absolute lateral position (≥ 100 cm) which occur within a window of 8 s did occur frequently and were able to demonstrate treatment-induced driving impairment in most conditions. However, the measure seems to be a simple transformation of the SDLP with inferior sensitivity to treatment-induced driver impairment. Also, the detection rate is biased in the direction of drivers with higher inherent SDLP values. It is therefore concluded that this outcome has little or no additional value to the SDLP as an outcome measure of the on-the-road driving test.
